# Are YouTube Videos on Tarsal Tunnel Syndrome Reliable? An In‐Depth Assessment of Educational Content, Source, and Audience Engagement

**DOI:** 10.1002/jfa2.70180

**Published:** 2026-07-18

**Authors:** Ryan H. Nishi, Jenna Tsuzaki, Shashi Sharma, Matthew Kao, Cameron Nishida, Kyle K. Obana, Julian Rimm, Kyle Ishikawa, Kore Liow, Enrique Carrazana

**Affiliations:** ^1^ John A. Burns School of Medicine University of Hawaii Honolulu Hawaii USA; ^2^ ALS and Neuromuscular Center and Neuromuscular Research Unit Hawaii Pacific Neuroscience Honolulu Hawaii USA; ^3^ Department of Orthopedic Surgery Columbia University Irving Medical Center New York New York USA; ^4^ Department of Orthopedic Surgery University of Hawaii John A. Burns School of Medicine Honolulu Hawaii USA

**Keywords:** compression neuropathy, educational quality, tarsal tunnel syndrome, video analysis, YouTube

## Abstract

**Background:**

Tarsal tunnel syndrome (TTS) is a relatively uncommon compressive neuropathy of the posterior tibial nerve that is often underdiagnosed due to variable presentation and overlap with other conditions. As patients increasingly turn to YouTube for health information, concerns have arisen about the reliability and comprehensibility of such content.

**Methods:**

On December 23, 2024, YouTube was queried using the terms “tarsal tunnel syndrome,” “tarsal tunnel release,” and “tarsal tunnel injection.” After excluding duplicates, unrelated, non‐English, and short (< 30 seconds) videos, 88 were reviewed and the 50 most‐viewed were analyzed. Each was evaluated using three tools: the *Journal of the American Medical Association* (*JAMA*) benchmark criteria (0–4) for reliability, a 4‐point Likert scale for comprehensibility, and a 19‐point Tarsal Tunnel Syndrome‐Specific Score (TTS‐SS) for educational content. Video source, content type, and video power index (VPI) were also recorded. The Shapiro–Wilk test assessed normality, and Kruskal–Wallis and Wilcoxon rank sum tests compared group with Holm adjustment.

**Results:**

The median TTS‐SS educational content score was 5.2 (IQR 3.0–8.0). Mean *JAMA* and comprehensibility scores were 2.0 and 2.6, respectively. Only 10% of videos met all four *JAMA* criteria, and fewer than 20% were rated as highly comprehensible. Disease‐specific videos had the highest TTS‐SS (mean = 6.8), while non‐surgical management scored lowest (mean = 2.8, *p* = 0.025). Videos by trainers and physical therapists had higher VPI (mean = 338.4) than those by physicians (mean = 0.26, *p* < 0.001), despite lower educational value. Surgical technique videos were least comprehensible (mean = 1.4) compared with other types (*p* < 0.05).

**Conclusion:**

Among the most‐viewed English‐language YouTube videos sampled at a single time point, content related to TTS demonstrated generally low educational completeness, reliability, and comprehensibility. While healthcare professionals produce more accurate content, their videos attract less engagement than those by non‐physicians. Improving visibility of evidence‐based videos is necessary to ensure patients receive accurate information for this underrecognized condition.

## 1 Introduction

Tarsal tunnel syndrome (TTS) is a compressive neuropathy of the posterior tibial nerve as it passes through the fibro‐osseous tarsal tunnel beneath the flexor retinaculum on the medial aspect of the ankle [1]. Symptoms of TTS commonly include pain, numbness, and tingling, and can present similarly to other conditions such as plantar fasciitis, diabetic peripheral neuropathy, and radiculopathy [2]. Consequently, TTS remains largely underdiagnosed, and ongoing debate persists regarding optimal conservative management, timing of surgical intervention, best surgical approach, and management of recurrences [3, 4].

Although less common than other entrapment neuropathies, TTS is the fifth most commonly published entrapment syndrome in the literature [3]. Despite the wealth of scholarly literature on TTS, many patients turn to online platforms, including YouTube, for health information. A 2012 study found that 59% of U.S. adults searched the internet for medical information in the previous year [5], and more recent estimates suggest that approximately 75% of internet users perform online searches related to health topics [6], underscoring the growing reliance on digital platforms for medical information. The accessibility of online health information is closely tied to health literacy. Organizations including the National Institute of Health, the U.S. Department of Health and Human Services, and the American Medical Association recommend that patient education materials be written at approximately a sixth‐grade reading level to ensure comprehension by the general public [7]. When health information exceeds this level of complexity, patients may have difficulty understanding medical terminology or applying the information to their own care. YouTube, a widely utilized platform for sharing content that often lacks peer review, was accessed by 73% of adults in the United States and 91% of individuals aged 15 to 29 during the 2019–2020 period [8]. While YouTube offers convenience and accessibility, the quality of its content remains highly variable, and prior studies have documented inaccuracies in online health information, which may contribute to patient confusion and inappropriate self‐management [9]. This platform was selected for analysis because it is the largest publicly accessible video‐sharing platform and is widely used for dissemination of health‐related information. Prior studies evaluating patient education for musculoskeletal and orthopedic conditions have similarly focused on YouTube due to its high user engagement and accessibility to the general public. YouTube remains one of the most widely utilized sources of online health information worldwide, with more than two billion monthly users [10]. Prior studies have demonstrated substantial growth in the consumption of health‐related video content, particularly following the COVID‐19 pandemic, highlighting the increasing role of online platforms in patient education and healthcare decision‐making.

Despite a growing body of research on YouTube's role in patient education, no previous studies have specifically examined the quality, reliability, and comprehensibility of YouTube videos related to TTS. As patients increasingly turn to digital platforms for self‐education on medical conditions, YouTube has emerged as a widely used resource. Studies indicate that a substantial proportion of the public utilizes YouTube to gain knowledge about various health topics, making it essential to evaluate the quality of this content, especially when diagnosing complex conditions like TTS [11]. The reliance on online resources became particularly evident during the COVID‐19 pandemic, when both patients and healthcare professionals increasingly turned to internet‐based resources for health information [12]. Several studies have evaluated the quality of YouTube videos for other medical conditions, revealing consistent concerns about the reliability and comprehensiveness of online content. For instance, a study evaluating videos related to carpal tunnel syndrome [13] found that many videos reinforced misconceptions and failed to meet basic standards for medical transparency, with the average *JAMA* score significantly lower than expected. Similarly, a review of ulnar collateral ligament injuries on YouTube revealed a similar trend, where most videos lacked critical diagnostic and treatment information. These findings highlight a broader issue in online health education, where the quality of information is often compromised, even for commonly discussed orthopedic conditions.

Due to the diagnostic complexities of TTS and its overlap with other neuropathies, YouTube videos may inadvertently spread speculative treatments or misdiagnoses, making it essential to assess the quality of such online resources specific to TTS. As patients increasingly rely on such sources for medical guidance, it is imperative for healthcare providers to understand the quality and clarity of the information being disseminated. This study is the first to examine the educational quality, comprehensibility, and reliability of YouTube videos related to TTS, filling an important gap in the existing literature. When patients are well informed about the causes, diagnosis, and treatment of their condition, they are better able to participate in shared decision‐making and adhere to recommended management strategies [14]. Therefore, this study aimed to evaluate the reliability, educational quality, and comprehensibility of YouTube videos related to TTS.

## 2 Methods

This cross‐sectional observational study evaluated the reliability, educational content, and comprehensibility of YouTube videos related to TTS. The YouTube platform was searched on December 23, 2024, to compile a list of the most‐viewed videos relating to TTS. This search was conducted using Google Chrome in incognito mode while logged out of any Google or YouTube account to minimize personalization effects. The country setting was the United States, and the interface language was English. Videos were retrieved using YouTube's default sorting algorithm (relevance). For each search term, the first 50 videos returned were recorded, and duplicate videos appearing across search queries were removed before final selection. All videos included in the final dataset remained publicly available at the time of analysis.

Three independent searches were conducted using the key words, “tarsal tunnel syndrome,” “tarsal tunnel release,” “tarsal tunnel injection.” The videos were subsequently recorded and videos were excluded on the following exclusion criteria: videos unrelated to TTS, duplicate videos, videos not in English, and videos shorter than 30 seconds. A total of 88 videos were watched and recorded. Among these videos, 50 were analyzed based on the videos with the highest number of views. The rationale for analyzing a subset of the total videos was justified by the notion that most viewers only watch videos within the first few pages of results. This methodology is used in similar YouTube educational content videos [11, 12, 14].

### 2.1 Video Analysis

Basic measures of viewership and interaction with the videos were recorded including the duration, date uploaded, number of views, number of “likes,” views per day, likes per day, number of “dislikes,” and video source. Video power index (VPI) was calculated using the formula: VPI = (like ratio × view ratio)/100.

Video source was categorized into one of the following groups: academics, physicians, health professionals (nonphysician), trainers/physical therapists, medical sources, patients, and commercial. Academics were defined as videos produced by universities, a medical school, or affiliated research institutions that were clearly labeled as coming from academic programs. Videos created by physicians or any other health professionals at an academic institution were not counted as an academic source. Medical sources were defined as videos uploaded by hospitals, clinics, or healthcare systems presenting content without a clearly identified physician or academic affiliation. Commercial sources were defined as those created by companies or organizations promoting specific products, devices, or services, often with promotional intent. Video content was further classified by primary content type: exercise training, disease‐specific information, patient experience, surgical technique, nonsurgical management, and advertisement.

### 2.2 Quality Assessment

Prior to initiation of the study, 7 videos were randomly selected and watched independently by authors J.T., S.S., and R.N., as a calibration phase to create additional criteria for grading and discuss potential points of misinterpretation. Each video was independently reviewed by two evaluators J.T. and S.S. Any discrepancy in scoring between viewers was mediated through discussion with author R.N. Formal inter‐rater reliability statistics were not calculated, as consensus scoring was used to determine the final values included in the analysis. Three scoring systems were used. *JAMA* Benchmark Criteria was established by the *Journal of American Medical Association* and is used to assess the transparency and accountability of health‐related information sources [15, 17] (Table 1). This tool evaluates four core elements: authorship (identification of authors and their credentials), attribution (references and sources cited), disclosure (conflicts of interest or sponsorship clearly stated), and currency (dates of content creation or last update provided). Each element present receives one point, resulting in a score ranging from 0 to 4. For the *JAMA* authorship criterion, a point was awarded only when the video creator's full name and professional credentials (e.g., *John Smith, MD*) were explicitly provided. Usernames or partial identifiers (e.g., *Footsurgeon*, *DrJohnSmith*) without verifiable credentials were not considered sufficient. All videos automatically met the “currency” criterion, as YouTube provides the upload date by default. *JAMA* criteria have been widely used in previous studies evaluating online health information, including analyses of YouTube content for conditions such as carpal tunnel syndrome, rotator cuff pathology and knee arthroplasty [16, 17, 18]. Higher *JAMA* scores are considered to reflect greater reliability and adherence to standards of medical content transparency. However, *JAMA* criteria do not directly assess the accuracy or educational completeness of the content, making it most useful as a measure of source credibility and quality assurance practices.

**TABLE 1:** A description of the four *JAMA* benchmark criteria used to assess the reliability and transparency of health‐related video content.

Criterion	Description
Authorship	Author and contributor credentials and their affiliations should be provided
Attribution	All copyright information should be clearly listed, and references and sources for content should be stated
Currency	The initial date of posted content and dates of subsequent updates to content should be provided
Disclosure	Conflicts of interest, funding, sponsorship, advertising, support and video ownership should be fully disclosed

The comprehensibility score was a subjective 4‐point Likert scale adapted from prior studies to assess how understandable the video was for a general audience (1 = poor, 4 = excellent) (Table 2). Lastly, the Tarsal Tunnel Syndrome‐Specific Score (TTS‐SS) was adapted from previous studies focusing on the quality of YouTube content related to nerve compression [16, 18] (Table 3). The individual criteria were selected to reflect key elements of TTS commonly described in the clinical literature, including core aspects of presentation, diagnostic evaluation, and management. These domains were designed to capture whether videos addressed standard clinical features such as symptoms, risk factors, physical examination findings, diagnostic testing, and both operative and nonoperative treatment approaches. Each criterion was assigned one point to provide a structured measure of the completeness of educational information presented in each video. The total maximum TTS‐SS score was set at 19.

**TABLE 2:** Four‐point Likert scale used to assess the use of medical jargon and the accessibility of video content for a general audience.

1	Heavy use of medical jargon
2	Moderate use of medical jargon
3	Minimal use of medical jargon
4	Absent or negligible use of medical jargon

**TABLE 3:** List of 19 content‐specific criteria used to evaluate the educational value of each video related to tarsal tunnel syndrome.

Heading	Criteria
Presentation	Describes symptoms
	Describes patient population
General information	Describes tarsal tunnel anatomy
	Describes tarsal tunnel syndrome etiology
	Describes risk factors
Diagnosis and evaluation	Mentions physical examination findings
	Mentions electrophysiological tests
	Mentions the role of imaging
	Discusses differential diagnosis
Treatment	Mentions nonoperative treatment
	Mentions pharmacotherapy operative treatment
	Mentions operative treatment
	Mentions the indication of operative treatment
	Mentions open surgical technique
	Mentions endoscopic surgical technique
Postoperative care	Outlines recovery period
	Mentions need for post‐operative physical therapy
	Describes complications
	Describes outcomes

### 2.3 Ethics

This study was exempt from institutional review board (IRB) approval as it did not involve human or animal subjects. All analyses were conducted on publicly available, non‐identifiable online video content, in accordance with ethical standards and journal guidelines.

### 2.4 Statistics

The normality of each review score was assessed using the Shapiro–Wilk test. For normally distributed scores, parametric tests were employed: ANOVA to determine overall group differences and Student's *t*‐test for post‐hoc pairwise comparisons. Otherwise, non‐parametric tests were utilized: the Kruskal–Wallis rank sum test for overall differences and the Wilcoxon rank sum test for post‐hoc pairwise comparisons. Only the TTS specific score was normally distributed within all content category groups. All post‐hoc pairwise comparisons used the Holm method for *p*‐value adjustment. All analyses were conducted using R version 4.5.0.

## 3 Results

The top 50 videos with the most amount of views were included in this study. The median number of views was 34,090 (IQR 13,154–61,762), and the median duration was 13,200 seconds (IQR 8,880–29,640) (Table 4). The mean *JAMA* score, comprehensibility score and TTS‐SS were 2, 2.6, and 5, respectively. The majority of videos were uploaded by physicians (36%), trainers or physical therapists (28%), and other health professionals (24%) (Table 5). The remaining videos were produced by academics (8%), patients (2%), and miscellaneous medical sources (2%).

**TABLE 4:** Median values and interquartile ranges for video duration, number of views, engagement metrics, and overall scores for reliability (*JAMA*), comprehensibility and TTS‐SS.

Characteristic	Median (Q1–Q3)
Video duration (min)	3.7 (2.5–8.2)
Number of views	34,090 (13,154–61,762)
Number of likes	248 (65–668)
Number of dislikes	10 (2–18)
Video power index	0.31 (0.02–2.5)
*JAMA* score (0–4)	2.0 (2–3)
Comprehensibility score (0–4)	2.6 (2–4)
TTS‐SS score (0–19)	5.2 (3.0–7.0)

**TABLE 5:** Frequency and percentage of videos categorized by source.

Video source	Count	Percentage
Physicians	18	36
Trainers/physical therapists	14	28
Health professionals	12	24
Academics	4	8
Patients	1	2
Medical sources	1	2

Most videos (48%) focused on disease‐specific information, while others emphasized exercise training (16%), surgical technique (14%), patient experiences (12%), or non‐surgical management (10%). When comparing video content by source, trainers and physical therapists produced videos with significantly higher video power indices (mean = 338.4) than physicians (mean = 0.26, *p* < 0.001). Similarly, exercise training videos demonstrated higher video power indices than both surgical technique (*p* = 0.034) and patient experience videos (*p* = 0.013).

Quality assessment using the *JAMA* benchmark criteria revealed that only 10% of videos fulfilled all four benchmarks, while 78% met two (Table 6). The mean *JAMA* score was higher among videos created by physicians (2.2) and health professionals (2.1) than those by other sources. The mean comprehensibility score for all videos was 2.6 (SD 1.1). Surgical technique videos were less comprehensible (mean = 1.4) than non‐surgical management (3.2, *p* = 0.037) and exercise training videos (3.5, *p* = 0.021).

**TABLE 6:** Frequency and percentage of videos meeting 1, 2, 3, or all 4 *JAMA* benchmark criteria for reliability.

*JAMA* score	Count	Percentage
1	5	10
2	39	78
3	5	10
4	1	2

The median total Tarsal Tunnel Syndrome‐Specific Score (TTS‐SS) across all videos was 5.0 (IQR 3.0–8.0), with a maximum possible score of 19 (Table 4). Videos focusing on disease‐specific information scored highest (mean = 6.8), whereas those addressing non‐surgical management scored lowest (mean = 2.8, *p* = 0.025) (Table 7). There was no statistically significant difference in TTS‐SS by video source after adjustment for multiple comparisons (Table 8). Post hoc analyses were performed by aggregating underrepresented video sources (academics, patients, and medical sources) into an “other” category, and consolidating content categories to improve statistical power. These analyses reinforced key findings. Videos produced by trainers or physical therapists demonstrated a markedly higher video power index (mean = 338.4) than those by physicians (mean = 0.26, *p* < 0.001), indicating substantially greater audience engagement. In terms of content type, exercise training videos had a higher video power index (mean = 23.6) compared to patient experience (mean = 0.05, *p* = 0.013) and surgical technique videos (mean = 0.11, *p* = 0.034). Similarly, disease‐specific videos showed greater engagement (mean = 201.4) than patient experience videos (*p* = 0.034). Surgical technique videos also had significantly lower comprehensibility scores (mean = 1.4) compared to non‐surgical management (mean = 3.2, *p* = 0.037) and exercise training videos (mean = 3.5, *p* = 0.021). Together, these findings suggest that physical therapist‐generated and disease‐specific content is associated with both higher educational value and viewer engagement, while surgical content tends to be less accessible to a general audience.

**TABLE 7:** Frequency and percentage of videos categorized by primary content type.

Content type	Count	Percentage
Disease‐specific information	24	48
Exercise training	8	16
Surgical technique	7	14
Patient experience	6	12
Non‐surgical management	5	10

**TABLE 8:** Mean values and standard deviations for video power index, *JAMA* score, comprehensibility score, and TTS‐SS, stratified by video source and content type.

Characteristic	Video power index		*JAMA* benchmark criteria		Video comprehensibility		Total TTS specific score	
	*N* = 50^a^	*p*‐value^b^	*N* = 50^c^	*p*‐value^b^	*N* = 50^d^	*p*‐value^b^	*N* = 50^e^	*p*‐value^f^
Video source		0.003		0.151		0.110		0.215
Academics	5.37 (6.28)		1.8 (0.5)		2.0 (0.0)		6.0 (6.8)	
Health professionals	21.59 (63.41)		2.1 (0.3)		3.0 (0.7)		7.2 (3.9)	
Medical sources	0.00 (NA)		1.0 (NA)		1.0 (NA)		0.0 (NA)	
Patients	0.00 (NA)		2.0 (NA)		4.0 (NA)		6.0 (NA)	
Physicians	0.26 (0.45)		2.2 (0.4)		2.4 (1.1)		5.4 (3.4)	
Trainers/physical therapist	338.40 (1,193.56)		2.0 (0.8)		2.8 (1.1)		4.6 (2.8)	
Exercise		< 0.001		0.864		0.001		0.025
Disease‐specific information	201.35 (913.26)		2.1 (0.7)		2.5 (0.9)		6.8 (4.4)	
Non‐surgical management	0.17 (0.22)		2.0 (0.0)		3.2 (0.4)		2.8 (2.0)	
Patient experience	0.05 (0.09)		2.0 (0.0)		3.0 (1.1)		6.5 (3.4)	
Surgical technique	0.11 (0.17)		2.1 (0.4)		1.4 (0.8)		5.0 (2.2)	
Training	23.58 (41.72)		1.9 (0.4)		3.5 (0.8)		3.4 (1.3)	

^a^ Video power index: Mean (SD).

^b^ Kruskal–Wallis rank sum test.

^c^ *JAMA* benchmark criteria: Mean (SD).

^d^ Video comprehensibility: Mean (SD).

^e^ Total TTS‐SS score: Mean (SD).

^f^ Kruskal–Wallis rank sum test; one‐way analysis of means (not assuming equal variances).

## 4 Discussion

This study is the first to systematically evaluate the quality, reliability, and comprehensibility of YouTube videos related to tarsal tunnel syndrome (TTS). Because TTS frequently presents with symptoms that overlap with plantar fasciitis, diabetic peripheral neuropathy, lumbar radiculopathy, and other lower‐extremity disorders, access to accurate educational resources is particularly important for patient understanding and timely referral. Our findings demonstrate that the overall educational value of TTS‐related videos is poor. Only 10% of videos met all *JAMA* benchmark criteria, fewer than 20% were rated as highly comprehensible, and the mean Tarsal Tunnel Syndrome‐Specific Score (TTS‐SS) was just 5.2 out of a possible 19. While disease‐specific videos offered somewhat higher educational content, videos created by trainers and physical therapists—despite generating substantially higher engagement—were generally less reliable and scored lower in content quality.

These results align with prior studies evaluating YouTube as a source of medical education for other orthopedic and neurological conditions. Analyses of carpal tunnel syndrome [13], cubital tunnel syndrome [19], and ulnar collateral ligament injuries [20] similarly demonstrated low reliability, inconsistent comprehensibility, and frequent omission of essential diagnostic or treatment information. Our finding that surgical technique videos were among the least comprehensible mirrors prior studies of carpal tunnel and scoliosis content [19, 21], underscoring that technically complex information is rarely presented in a way that is accessible to the general public. These findings are also consistent with broader evaluations of YouTube content across orthopedic [22, 23, 24], musculoskeletal [25], and general health‐related domains [26], which similarly documented substantial variability in reliability and educational quality.

A unique challenge in this study was applying *JAMA* benchmark criteria to YouTube videos. Authorship is difficult to evaluate given the variety of ways creators identify themselves. Although we required both a full name and professional credentials for a point, many videos only listed usernames or partial names that did not meet this threshold. As a result, authorship scores were consistently low. In contrast, all videos automatically fulfilled the “currency” criterion since YouTube displays upload dates, which may artificially elevate *JAMA* scores compared with other online resources where publication or update dates are often absent.

Beyond the mechanics of *JAMA* scoring, the platform itself poses structural limitations for evaluating reliability. Unlike PubMed‐indexed literature, YouTube videos are not subject to peer review. Any individual can upload content regardless of accuracy or expertise, leaving audiences unable to easily distinguish credible medical information from personal opinion or misinformation. The lack of editorial oversight is a fundamental contrast between YouTube and peer‐reviewed journals.

Another concern is the absence of disclosure requirements on YouTube. Many content creators and influencers receive compensation to promote products, devices, or therapies, yet such financial relationships are rarely acknowledged. The commercial influence in this space is substantial: global influencer marketing expenditures have increased from approximately $9.7 billion in 2020 to a projected $32.6 billion in 2025, reflecting the rapid growth of this industry and its potential to shape online health content without transparency of conflicts of interest [27].

Finally, YouTube's accessibility is both a strength and a liability. Its free, widely available format enables rapid dissemination of health information to a global audience. However, the same features magnify the spread of inaccurate or promotional material, particularly because algorithms prioritize engagement over educational quality. This imbalance, high reach but low reliability, underscores the need for clinicians to be aware of the content their patients may encounter and, when possible, to guide them toward trustworthy educational resources.

The consequences of poor‐quality online information extend beyond simple misunderstanding [28]. Inaccurate or incomplete educational materials may alter patient perceptions regarding disease severity, influence expectations of treatment outcomes, delay presentation to healthcare professionals, and contribute to inappropriate self‐management strategies. These concerns may be particularly relevant in TTS, where diagnostic uncertainty already contributes to underrecognition and delayed treatment.

Because individuals with diverse educational, cultural, and socioeconomic backgrounds access online health information, digital educational materials must be designed to be understandable to a broad audience. In the present study, fewer than 20% of videos were rated as highly comprehensible, highlighting a potential barrier to patient education. Prior work evaluating AI‐generated health information has similarly emphasized that clear and simple language is essential for helping patients accurately interpret disease processes and treatment options. Educational resources that rely heavily on medical terminology or assume advanced health literacy may unintentionally limit patient understanding and contribute to misinformation or inappropriate healthcare decisions. Therefore, improving readability and accessibility should be considered a fundamental component of online patient education alongside accuracy and reliability [29].

Beyond platform‐specific engagement, the dissemination of medical information is increasingly evaluated using bibliometric and alternative metrics [30]. Traditional citation‐based measures may underestimate the real‐time impact of research, particularly in the digital era where social media and online platforms play a major role in knowledge dissemination. Bibliometric analyses incorporating Altmetric data provide insight into how frequently research is discussed, shared, and engaged with across online platforms. Prior work examining the most cited articles in musculoskeletal disease demonstrates that social media visibility and online engagement can complement traditional academic impact and enhance the reach of scientific literature. In this context, the high engagement observed in certain YouTube videos, despite lower educational quality, highlights a disconnect between academic rigor and public visibility. Bridging this gap remains an important challenge for clinicians and researchers aiming to improve patient education.

## 5 Strengths

This study has several strengths. To our knowledge, it is the first study to systematically evaluate the reliability, educational completeness, and comprehensibility of YouTube videos related specifically to tarsal tunnel syndrome. The analysis incorporated multiple complementary evaluation tools, including the *JAMA* benchmark criteria, a comprehensibility Likert scale, and the Tarsal Tunnel Syndrome‐Specific Score (TTS‐SS), allowing for a multidimensional assessment of video content. Additionally, the use of a structured, condition‐specific scoring system enabled a focused evaluation of whether videos addressed clinically relevant aspects of TTS, including presentation, diagnostic evaluation, treatment options, and postoperative considerations. Together, these approaches provide a more comprehensive assessment of the educational value of online video content related to this condition.

## 6 Limitations

This study has several limitations. First, the application of *JAMA* benchmark criteria to YouTube is imperfect. Authorship is difficult to verify given the wide range of identifiers used by video creators. Furthermore, because upload dates are automatically displayed, all videos meet the “currency” criterion, which artificially inflates scores relative to other online platforms. Second, YouTube lacks a peer‐review process. In contrast to scholarly literature, content can be posted by anyone regardless of medical training, making it difficult to ensure accuracy. The absence of editorial oversight is a fundamental limitation of using this platform for patient education research. Third, conflicts of interest are rarely disclosed on YouTube, despite the growing scale of commercial influence. With influencer marketing expenditures increasing more than tenfold in five years, undisclosed financial incentives may bias the information presented in health‐related videos. Fourth, this study included only English‐language content and was limited to the 50 most‐viewed videos. While this reflects typical patient behavior, it may exclude less‐viewed or non‐English videos that provide higher quality information. Finally, although we performed a calibration phase to standardize evaluations, the comprehensibility score remained subjective, and formal inter‐rater reliability testing was not conducted. Although a calibration phase and consensus review process were used, formal inter‐rater reliability statistics were not calculated, which may limit the ability to quantify agreement between evaluators. The TTS‐SS was designed to reward objective features of educational quality, but it has not undergone external validation.

## 7 Future Directions

Future research should focus on validating scoring tools like the TTS‐SS and assessing inter‐rater reliability. Automated algorithms for detecting misleading or incomplete medical information on video platforms may offer scalable solutions. In parallel, professional societies could develop curated YouTube playlists or partner with skilled content creators to improve the visibility and engagement of high‐quality educational content. Policy‐level interventions, such as requiring medical disclosure tags or content verification for health‐related videos, may also warrant exploration.

In addition to video‐sharing platforms such as YouTube, patients increasingly obtain health information from artificial intelligence‐based tools and large language model platforms, including ChatGPT, Gemini, and Perplexity AI. Unlike YouTube, which relies on user‐generated video content that may reflect personal experience or promotional intent, AI‐based platforms generate structured, text‐based responses derived from large‐scale training data. This distinction introduces different strengths and limitations. AI‐generated responses may provide more standardized and comprehensive answers to common medical questions; however, their accuracy, readability, and reliability remain variable and are dependent on model training, prompt structure, and source data. Emerging evidence suggests that AI‐based tools may also provide exercise recommendations, rehabilitation guidance, and educational information for chronic musculoskeletal and neurologic conditions [31]. These technologies may help bridge gaps in patient access to medical information by providing personalized and on‐demand responses. However, the quality, accuracy and reliability of such recommendations remain variable and require continued evaluation.

Recent studies evaluating AI‐generated medical content have demonstrated variability in readability and quality, with some platforms producing information that is more accessible but not consistently aligned with evidence‐based standards [32, 33]. In contrast, YouTube content is highly variable in both accuracy and presentation, with engagement often driven by entertainment value rather than educational quality. These differences highlight that while AI platforms may improve accessibility and structure of information, they do not eliminate concerns regarding accuracy and reliability. For patients with TTS, AI‐based tools may eventually provide guidance regarding symptom recognition, conservative treatment options, activity modification, and post‐operative recovery expectations [31]. Future studies should evaluate the accuracy and comprehensiveness of AI‐generated educational content for TTS and compare these outputs directly with information available on video‐sharing platforms.

As digital health information continues to evolve, future research should aim to directly compare the quality, reliability, and patient comprehension of information obtained from AI‐based tools and traditional online platforms such as YouTube. Understanding these differences will be critical in guiding patients toward accurate and accessible sources of medical information.

## 8 Conclusion

YouTube remains a widely used yet unregulated source of health information. For a condition like TTS, often underrecognized and misdiagnosed, the prevalence of low‐quality videos presents a barrier to patient education. While healthcare professionals tend to produce more accurate content, such videos are less likely to engage viewers. Bridging this gap will require a coordinated effort between clinicians, educators, and digital media creators to ensure that online information is not only accurate, but also accessible and engaging.

Although artificial intelligence platforms may improve accessibility and provide rapid responses to patient questions, current evidence suggests that these technologies should be viewed as complementary educational tools rather than substitutes for professional medical evaluation [34]. Critical aspects of diagnosis, individualized treatment planning, risk‐benefit assessment, and recognition of warning signs continue to require clinician oversight. Physical examination findings, individualized clinical decision‐making, and recognition of subtle diagnostic features remain essential components of patient care that cannot be fully replicated by digital platforms [35]. Therefore, these resources should be viewed as supplementary educational tools rather than substitutes for direct physician oversight and patient–physician interaction.

## Author Contributions

**Ryan H. Nishi:** conceptualization, methodology, investigation, data curation, writing – original draft, writing – review and editing, project administration. **Jenna Tsuzaki:** investigation, data curation, writing – original draft, writing – review and editing. **Shashi Sharma:** investigation, data curation, writing – original draft, writing – review and editing. **Matthew Kao:** investigation, data curation, writing – review and editing. **Cameron Nishida:** investigation, data curation, writing – review and editing. **Kyle K. Obana:** methodology, formal analysis, writing – review and editing, supervision. **Julian Rimm:** methodology, formal analysis, writing – review and editing. **Kyle Ishikawa:** formal analysis. **Kore Liow:** conceptualization, supervision, writing – review and editing. **Enrique Carrazana:** conceptualization, methodology, investigation, writing – review and editing.

## Funding

The authors have nothing to report.

## Conflicts of Interest

The authors declare no conflicts of interest.

## Data Availability

The data that support the findings of this study are available in YouTube at https://www.youtube.com/. These data were derived from the following resources available in the public domain: YouTube, https://www.youtube.com/.
